# Serum Insulin‐Like Growth Factor‐1 (IGF‐1) in Parkinson's Disease: A Systematic Review and Meta‐Analysis

**DOI:** 10.1002/brb3.71872

**Published:** 2026-11-05

**Authors:** Sajjad Mahdavi, Shahryar Rajai Firouzabadi, Ida Mohammadi, Soroush Oraee, Mohammadreza Alinejadfard, Seyed Ali Mofidi, Mehri Salari, Ehsan Sharifipour

**Affiliations:** ^1^ School of Medicine Shahid Beheshti University of Medical Sciences Tehran Iran; ^2^ Functional Neurosurgery Research Center, Shohada Tajrish Comprehensive Neurosurgical Center of Excellence Shahid Beheshti University of Medical Sciences Tehran Iran; ^3^ Shahid Beheshti University of Medical Sciences Tehran Iran

**Keywords:** IGF‐1, insulin‐like growth factor‐1, Parkinson's disease, primary parkinsonism, somatomedin C

## Abstract

**Background:**

Parkinson's disease (PD) is a common neurodegenerative disorder characterized by progressive motor and non‐motor symptoms. Insulin‐like Growth Factor‐1 (IGF‐1), a neuroprotective hormone, has been proposed as a potential biomarker in PD.

**Objectives:**

This study aimed to evaluate differences in serum IGF‐1 levels between people with PD (PwPD) and healthy controls, and to assess associations with symptom severity.

**Methods:**

We conducted a systematic search of PubMed, Embase, and Web of Science on December 7, 2024. Observational studies comparing serum IGF‐1 in PwPD and healthy controls, or assessing its associations with PD symptoms, were included. Study quality was evaluated using the Newcastle–Ottawa Scale. A random‐effects meta‐analysis pooled mean differences (MD) in IGF‐1 levels, and correlation coefficients were synthesized for motor symptoms. Non‐motor symptom associations were analyzed using effect direction plots.

**Results:**

Eighteen studies with 2,480 participants (1,465 PwPD; 1,015 controls) were included. PwPD had significantly higher serum IGF‐1 levels than controls (MD: 21.3 ng/mL; 95CI: 11.49, 31.12; *p <* 0.001). Elevated IGF‐1 was consistent across early/late‐stage and drug‐naïve/treated PwPD, with no significant subgroup differences. Meta‐analysis of five studies (413 PwPD) showed an inverse correlation between IGF‐1 levels and motor symptom severity (*r =* −0.35; 95% CI: −0.53, −0.08; *p <* 0.01), although individual study findings were heterogeneous, and the certainty of evidence was low. Non‐motor symptoms also showed no consistent associations.

**Conclusions:**

PwPD exhibit elevated serum IGF‐1 levels and may be inversely associated with motor symptom severity. IGF‐1's relationship with non‐motor symptoms remains unclear and requires further study. Longitudinal research is needed to determine IGF‐1's utility as a PD biomarker

## Graphical abstract

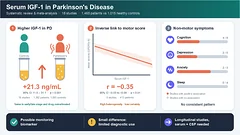

## 1 Introduction

Parkinson's disease (PD) is the second most common neurodegenerative disorder, affecting 1–2 per 1,000 people in the population (Tysnes and Storstein 2017). It is characterized by hallmark motor symptoms, including tremor, rigidity, and bradykinesia, as well as a wide range of non‐motor symptoms such as cognitive impairment, depression, and sleep disorders (Hayes 2019; Chaudhuri and Schapira 2009; Aarsland et al. 2021). PD is characterized by the progressive degeneration of dopaminergic neurons, particularly in the substantia nigra, likely due to the accumulation of misfolded phosphorylated α‐synuclein in Lewy bodies. This process is associated with mitochondrial dysfunction, oxidative stress, neuroinflammation, and lysosomal impairment; however, the precise mechanisms driving PD pathogenesis remain incompletely understood (Kouli et al. 2018). Given the challenges in early diagnosis and the need to monitor disease progression and therapeutic efficacy, identifying reliable biomarkers has become increasingly important. Several cerebrospinal fluid (CSF), plasma, and MRI biomarkers are being extensively investigated (Ryman and Poston 2020; He et al. 2018). However, current biomarkers for PD face challenges due to variability in study results, lack of disease specificity, and limited correlation with clinical symptoms, hindering their effectiveness in early diagnosis and monitoring disease progression (Li and Le 2020).

Insulin‐like Growth Factor‐1 (IGF‐1), a polypeptide hormone integral to neuronal growth, differentiation, and survival, has garnered attention as a potential biomarker for PD (Dyer et al. 2016). Although IGF‐1 is primarily produced by the liver for endocrine purposes, it is also synthesized locally in the brain by various cell types, including astrocytes, microglia, and neurons (Pristerà et al. 2019; Herrera et al. 2024). Previous studies have suggested IGF‐1 exerts neuroprotective effects in people with PD (PwPD) and may be elevated in these patients, possibly due to a compensatory mechanism in response to nerve injuries (Bhalla et al. 2022; Hayes et al. 2021). Alterations in IGF‐1 signaling pathways have also been documented in PwPD (Yang et al. 2018; Castilla‐Cortázar et al. 2020). Moreover, studies have shown that IGF‐1 is involved in promoting the survival of dopaminergic neurons and protecting them from toxin‐induced damage. It also modulates neuroinflammation, which is a key factor in PD progression (Herrera et al. 2024).

Despite these findings, the clinical relevance of serum IGF‐1 as a diagnostic or monitoring tool in PD remains uncertain, with studies reporting inconsistent results regarding its levels in PwPD compared to healthy controls and its associations with their clinical manifestations (Picillo et al. 2013; Godau et al. 2011). A previous meta‐analysis found higher serum IGF‐1 levels in PwPD compared to healthy controls (Alwerdani et al. 2022); however, no systematic review and meta‐analysis has previously evaluated the association between serum IGF‐1 levels and motor and non‐motor symptoms in PD.

Therefore, the primary objective of this systematic review and meta‐analysis was to assess whether serum IGF‐1 levels differ between PwPD and healthy controls. Additionally, we examined the associations between serum IGF‐1 L and the severity of motor symptoms, as well as their relationships with non‐motor symptoms, including depression, anxiety, cognitive function, and sleep disorder, in PwPD.

## 2 Methods

This study was carried out in accordance with the Preferred Reporting Items for Systematic Reviews and Meta‐Analyses (PRISMA) guidelines (Page et al. 2021) with prospective protocol registration in PROSPERO (CRD42025637056).

### 2.1 Search Strategy

A comprehensive literature search was performed on December 7, 2024, across PubMed, Embase, and Web of Science databases using keywords and MeSH terms including “Insulin‐Like Growth Factor 1”, “IGF‐1”, “Somatomedin C”, “Parkinson's Disease”, and “Primary Parkinsonism.” No language or publication year limitations were applied. Details of the search strategies for each database are available in Table S1.

### 2.2 Screening and Study Selection

Screening was performed in two phases by two independent reviewers (S.M. and S.R.F.) under the supervision of a third reviewer (E.S.). An initial title and abstract screening phase was followed by full‐text retrieval and evaluation. Any discrepancies were resolved via discussion. Papers were included if they met our predefined PECOS criteria: (P)opulation: human participants diagnosed with primary PD; (E)xposure: not applicable; (C)omparator: healthy controls (HC); (O)utcome: serum IGF‐1 levels in PwPD and HC participants or associations between motor and/or non‐motor symptoms and IGF‐1 levels in PwPD; (S)tudy design: observational cross‐sectional, case‐control, or longitudinal studies.

Exclusion criteria consisted of patients with atypical parkinsonism, interventional studies, case reports, case series, and conference abstracts. Studies with overlapping populations or those using similar datasets (such as PPMI) were identified, and the study with the larger dataset was included in the review. The PRISMA flowchart was used to depict the selection process using the tool developed by Haddaway et al. (2022).

### 2.3 Data Extraction

Data extraction was independently conducted by two reviewers (S.M. and S.R.F.) under the supervision of a third reviewer (E.S.) using pre‐constructed data sheets. Discrepancies were resolved via independent validation. The extracted data included the first author, year of publication, country of origin of the study population, study design (follow‐up duration in case of longitudinal design), source of data, fasting or non‐fasting serum sample, method of IGF‐1 detection (categorized into chemiluminescence immunoassay (CLIA), enzyme‐linked immunosorbent assay (ELISA), and immunoradiometric assay (IRMA)), number of PwPD and HC participants, drug‐naïve status of PwPD, disease stage of PwPD (early or late), disease duration (years), mean age, sex ratio (male%), adjustment for confounding factors, mean IGF‐1 levels and corresponding standard deviations in PwPD and HC participants, associations of IGF‐1 with motor symptoms, and associations of IGF‐1 with non‐motor symptoms. When results were reported graphically, quantitative values were obtained via PlotDigitizer (2024). All IGF‐1 concentrations were converted to ng/mL.

### 2.4 Outcomes

Our outcomes of interest included (Tysnes and Storstein 2017) the comparison of IGF‐1 levels between PwPD and HC participants, (Hayes 2019) the associations between IGF‐1 levels in PwPD and motor symptoms (assessed using the UPDRS‐III) and (Chaudhuri and Schapira 2009) the associations between IGF‐1 levels in PwPD and non‐motor symptoms (as defined in Lee and Koh (2015)). Non‐motor symptoms included neuropsychiatric symptoms, such as depression, anxiety, and cognitive dysfunction, alongside sleep disorders. For cognitive dysfunction, only global cognitive summary scores or tests designed for screening of cognitive dysfunction (i.e., MoCA, MMSE) were eligible.

### 2.5 Quality Assessment

To assess methodological quality, the Newcastle‐Ottawa Scale (NOS) was applied, which is a validated tool for evaluating observational studies, including cohort, case‐control, and cross‐sectional designs (Wells et al. 2014; Peterson et al. 2011). Two reviewers (S.M. and M.A.) assessed the included studies. Any conflicts were resolved through consultation with a third reviewer (E.S.). Each study design was evaluated using its specific Newcastle‐Ottawa Scale questionnaire, which assessed studies based on criteria such as selection, comparison, and exposure for case‐control studies, and selection, comparison, and outcome for cohort and cross‐sectional studies.

Finally, studies that did not receive appropriate ratings for at least three items were classified as low quality, whereas those that did were classified as high quality. A detailed schematic assessment of the quality of studies was created using the Risk of Bias visualization tool (ROBVIS) (McGuinness and Higgins 2021).

### 2.6 Certainty of Evidence Assessment

The certainty of evidence was evaluated using the GRADE (Grading of Recommendations, Assessment, Development, and Evaluation) framework. This system classifies evidence into four tiers. High certainty indicates that the true effect is likely to be close to the estimated effect. The next tier, moderate certainty, suggests that the actual effect is probably similar to the estimate, but some differences may exist. Low certainty indicates that the true effect might differ significantly from the estimate, while very low certainty means there is little trust in the estimate, and the actual effect could be substantially different (Guyatt et al. 2011).

Observational studies started with a low certainty rating, which could be adjusted upward or downward depending on specific criteria. Evidence was downgraded if there were issues such as bias, inconsistent results, indirectness, imprecise data, or potential publication bias. On the other hand, evidence could be upgraded if there was a notably strong effect, if confounding factors would only strengthen the observed effect, or if a dose‐response relationship was evident.

If key clinical studies were found to have a high risk of bias, the certainty in all related outcomes was reduced by one level. When the variation in results across studies was greater than 50%, the certainty of evidence was downgraded for significant inconsistency. If over 90% of the studies showed major inconsistencies, this was considered a very serious issue. Evidence directly addressing the research question was not penalized for indirectness. However, if confidence intervals were broad and included both clinically meaningful and non‐meaningful effects, the certainty of evidence was downgraded for serious imprecision. In cases where there were only a few studies for specific outcomes, publication bias could not be formally evaluated but was strongly suspected. Lastly, if the results did not demonstrate a large effect, plausible confounding, or a dose‐response relationship, the certainty of evidence was not upgraded.

### 2.7 Data Synthesis

Pairwise meta‐analyses were carried out using Stata version 18 (STATA Corporation, College Station, TX). The mean difference (MD) of serum IGF‐1 levels between PwPD and HC participants was pooled using a random effects model to account for the difference in populations’ age and method of IGF‐1 evaluation. A *p‐*value < 0.05 was considered significant. Heterogeneity was evaluated using the *I^2^* statistic with *I^2^* > 50% defined as significant heterogeneity (Higgins et al., 2021). In case of heterogeneity in the pairwise meta‐analysis, meta‐regressions were conducted with MD of age, sex ratio, and disease duration as confounders. Subgroup analysis was conducted according to early or late PD status and drug‐naïve status. In case one study used the same HC group to compare early and late PwPD, the number of HC participants was divided between the PD groups to avoid overcounting (Higgins et al., 2021). Subgroup difference was assessed using Pearson's chi‐squared test, with a *p‐*value < 0.1 considered significant (Richardson et al. 2019). Publication bias was evaluated by funnel plot symmetry and Egger's test. Sensitivity analysis was performed via the leave‐one‐out method.

Correlations between IGF‐1 levels in PwPD and motor symptoms were meta‐analyzed using the R software and the meta package. An inverse variance weighting was used to pool the studies with z‐transformation of correlation coefficients. A random effects model was used to account for methods of IGF‐1 evaluation. Effect sizes are reported as MD or pooled z‐correlation coefficients alongside their respective 95% confidence intervals (95% CI).

Associations between IGF‐1 levels in PwPD and non‐motor symptoms were synthesized using effect direction plots. This approach was utilized due to heterogeneous effect measures with no valid method for conversion, incomplete reporting of study outcomes, and incomplete reporting of *p‐*values. The findings of each study regarding each non‐motor symptom are presented as positive, negative, or conflicting associations. If one outcome was assessed using multiple scales of which 70% or more were in conformity with one another, the effect direction would be similar to the majority. If not, then conflicting associations would be reported (Boon and Thomson 2021).

## 3 Results

Our online database search yielded 484 articles, of which 256 remained for title and abstract screening after duplicate removal. 22 were chosen for full‐text retrieval, and 18 studies were included in this review (Picillo et al. 2013; Godau et al. 2011; Bernhard et al. 2016; Fan et al. 2020; Federico et al. 1999; Fiszer et al. 2010; Godau et al. 2010; Gu et al. 2024; Ma et al. 2015; Mashayekhi et al. 2010; Numao et al. 2014; Pellecchia et al. 2014; Picillo et al. 2017; Schaefer et al. 2008; Shi et al. 2023; Shi et al. 2022; Suzuki et al. 2019; Tuncel et al. 2009). Reasons for exclusion included overlapping populations (*n =* 1), populations including patients with diseases other than PD (*n =* 2), and interventional study design (*n =* 1) (Figure 1).

**FIGURE 1:**
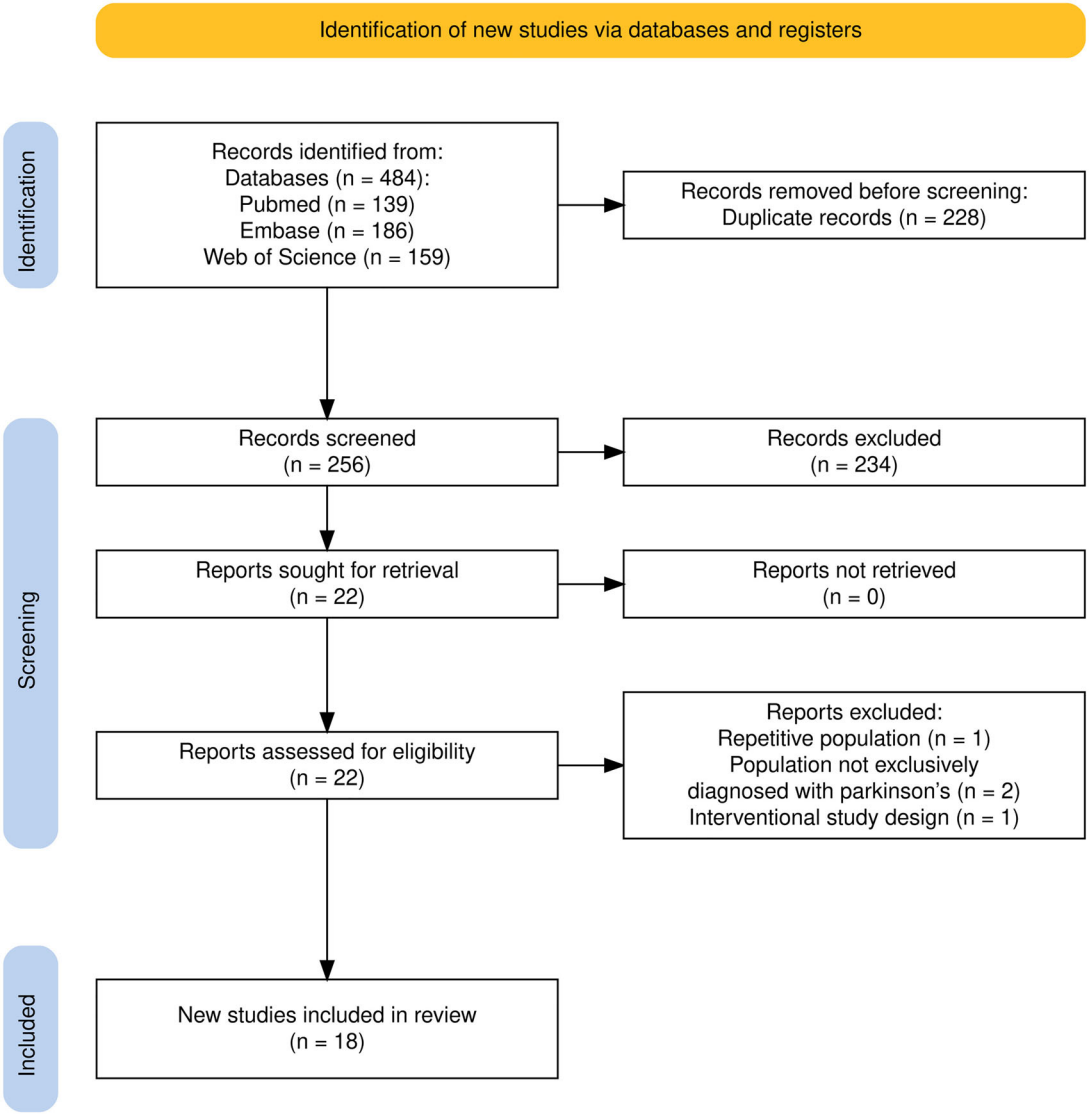
PRISMA flowchart detailing the literature search, screening, and selection process for the systematic review and meta‐analysis.

### 3.1 Characteristics of the Included Studies

Of the 18 studies, only 6 were published since 2018 (Fan et al. 2020; Gu et al. 2024; Schaefer et al. 2008; Shi et al. 2023; Shi et al. 2022; Suzuki et al. 2019). Four study populations originated from China (Gu et al. 2024; Ma et al. 2015; Shi et al. 2023; Shi et al. 2022), four from Germany (Godau et al. 2011; Bernhard et al. 2016; Godau et al. 2010; Schaefer et al. 2008), three from Italy (Picillo et al. 2013; Federico et al. 1999; Pellecchia et al. 2014), two from Japan (Numao et al. 2014; Suzuki et al. 2019), and one study each from New Zealand (Fan et al. 2020), Poland (Fiszer et al. 2010), Iran (Mashayekhi et al. 2010), and Turkey (Tuncel et al. 2009). One study used an international sample (Picillo et al. 2017). Ten of the studies were cross‐sectional in design, six were cohort studies, and two studies were case‐control studies. Sources of data were primarily local electronic medical records, with the exception of three studies that used longitudinal datasets (Godau et al. 2011; Bernhard et al. 2016; Picillo et al. 2017). 14 studies used fasting serum samples, with only two using non‐fasting samples (Fan et al. 2020; Suzuki et al. 2019) and two not mentioning fasting status (Fiszer et al. 2010; Mashayekhi et al. 2010). The method of testing IGF‐1 levels varied across studies, with six of the studies using ELISA (Picillo et al. 2013; Fan et al. 2020; Ma et al. 2015; Mashayekhi et al. 2010; Pellecchia et al. 2014; Picillo et al. 2017), six using CLIA (Godau et al. 2010; Gu et al. 2024; Schaefer et al. 2008; Shi et al. 2023; Shi et al. 2022; Tuncel et al. 2009), and six using IRMA (Godau et al. 2011; Bernhard et al. 2016; Federico et al. 1999; Fiszer et al. 2010; Numao et al. 2014; Suzuki et al. 2019) to measure IGF‐1.

A total of 2,480 participants consisting of 1,465 PwPD and 1,015 HC participants were included. Most studies used samples receiving medication, with only four studies using an exclusive drug‐naïve population (Picillo et al. 2013; Godau et al. 2011; Pellecchia et al. 2014; Picillo et al. 2017) and five studies using exclusively early stage PD samples (Picillo et al. 2013; Godau et al. 2011; Godau et al. 2010; Pellecchia et al. 2014; Picillo et al. 2017). Average disease duration was reported in 14 studies, with an average of 4.14 years since diagnosis. The average age and sex ratio of PwPD were reported in 17 studies, with a pooled mean age of 64.6 years and 55.86% male ratio. Of the seventeen studies with a control group, fourteen matched for age and sex while three had insufficient information. Further characteristics are available in Table S2. The outcomes assessed by each study are presented in Table S3, with their findings synthesized below.

### 3.2 Serum IGF‐1 in PwPD versus HC Participants

Seventeen studies comprising 1,392 PD and 1,015 HC participants compared IGF‐1 levels between the two groups (Picillo et al. 2013; Godau et al. 2011; Bernhard et al. 2016; Fan et al. 2020; Federico et al. 1999; Fiszer et al. 2010; Godau et al. 2010; Gu et al. 2024; Ma et al. 2015; Mashayekhi et al. 2010; Numao et al. 2014; Pellecchia et al. 2014; Picillo et al. 2017; Schaefer et al. 2008; Shi et al. 2023; Shi et al. 2022; Tuncel et al. 2009). All studies except the one by Federico et al. excluded or matched for IGF‐1 confounders. Data from 16 studies consisting of 1,382 PwPD and 1,005 HC participants were pooled and showed that PwPD had on average 21.3 ng/mL (95% CI: 11.49, 31.12, *p <* 0.001) higher serum IGF‐1 levels with high heterogeneity (*I^2^* = 85.82%; Figure 2) and low certainty (Table S4). No evidence of publication bias was detected in the funnel plot (Egger's *p‐*value = 0.259; Figure S1), and sensitivity analysis showed our results to be robust (Figure S2). The study by Schaefer et al. (2008) reported IGF‐1 levels as U/mL with no method of unit conversion and was therefore excluded from the meta‐analysis, yet they found an non‐significant increase of serum IGF‐1 in 10 PwPD compared to 10 age, sex and BMI matched controls (*p‐*value = 0.265), suggesting their findings are in line with our meta‐analysis.

**FIGURE 2:**
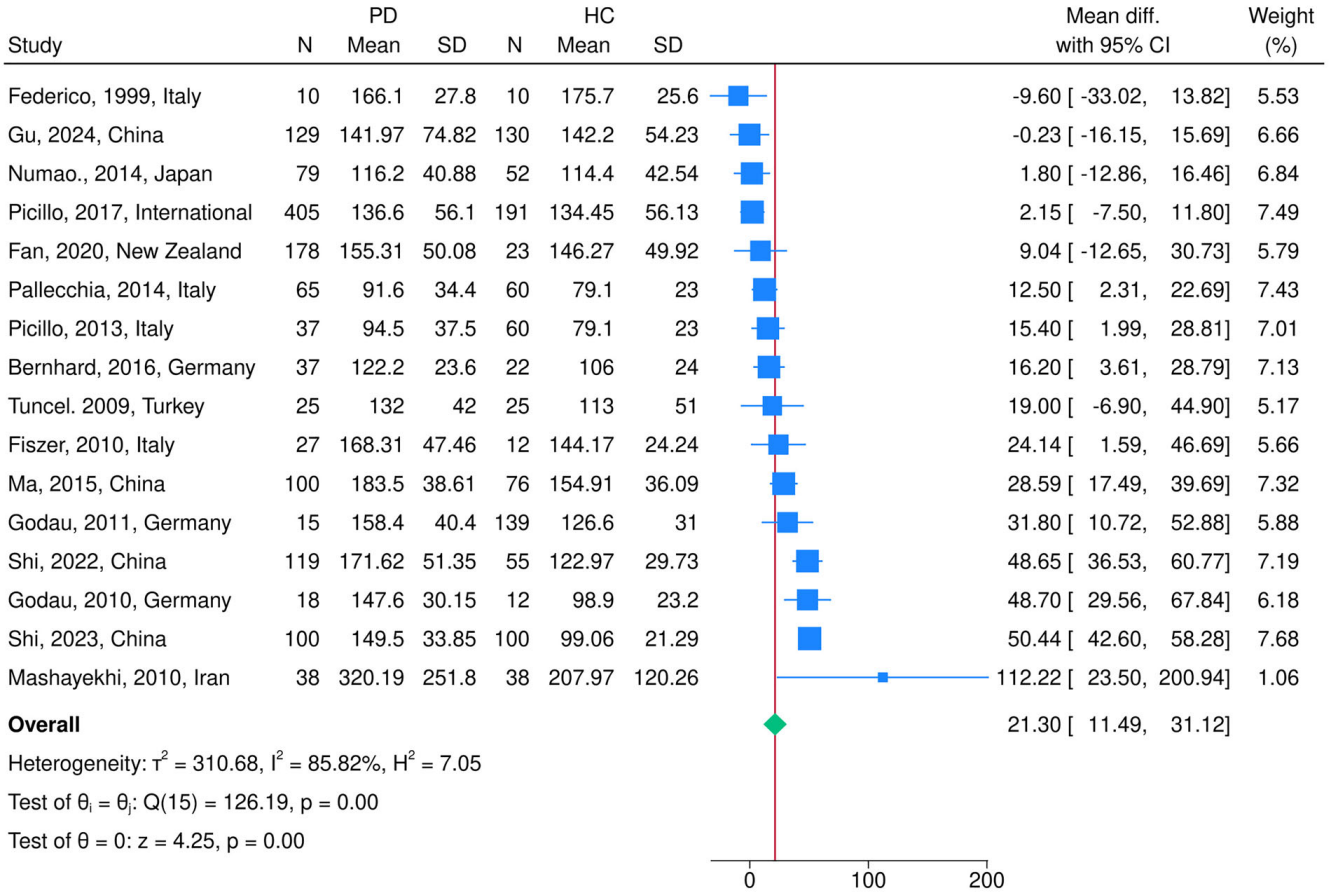
Pairwise meta‐analysis of serum IGF‐1 levels in patients with Parkinson's disease (PwPD) versus healthy controls (HC). The analysis was conducted using a random‐effects model with the effect measure expressed as mean difference (in ng/mL) (*I^2^* = 85.82%).

In order to investigate the heterogeneity, meta‐regressions were conducted for age difference, relative sex ratio, and disease duration (Table S5). None of these confounders showed a moderator effect. Subgroup analysis by early versus late‐stage PD showed both early stage (MD: 25.14 ng/mL; 95% CI: 9.92, 40.36, *p <* 0.001) and late stage (MD: 29.88 ng/mL; 95% CI: 17.78, 41.97, *p <* 0.001) PwPD had higher IGF‐1 levels compared to controls with no significant subgroup difference (*p‐*value = 0.63; Figure 3). Furthermore, drug‐naïve PwPD (MD: 18.09 ng/mL; 95% CI: 7.36, 28.82) and PwPD under treatment (MD: 30.72, 95% CI: 18.95, 42.49) both had higher IGF‐1 levels compared to controls with no significant subgroup difference (*p‐*value = 0.12; Figure 4).

**FIGURE 3:**
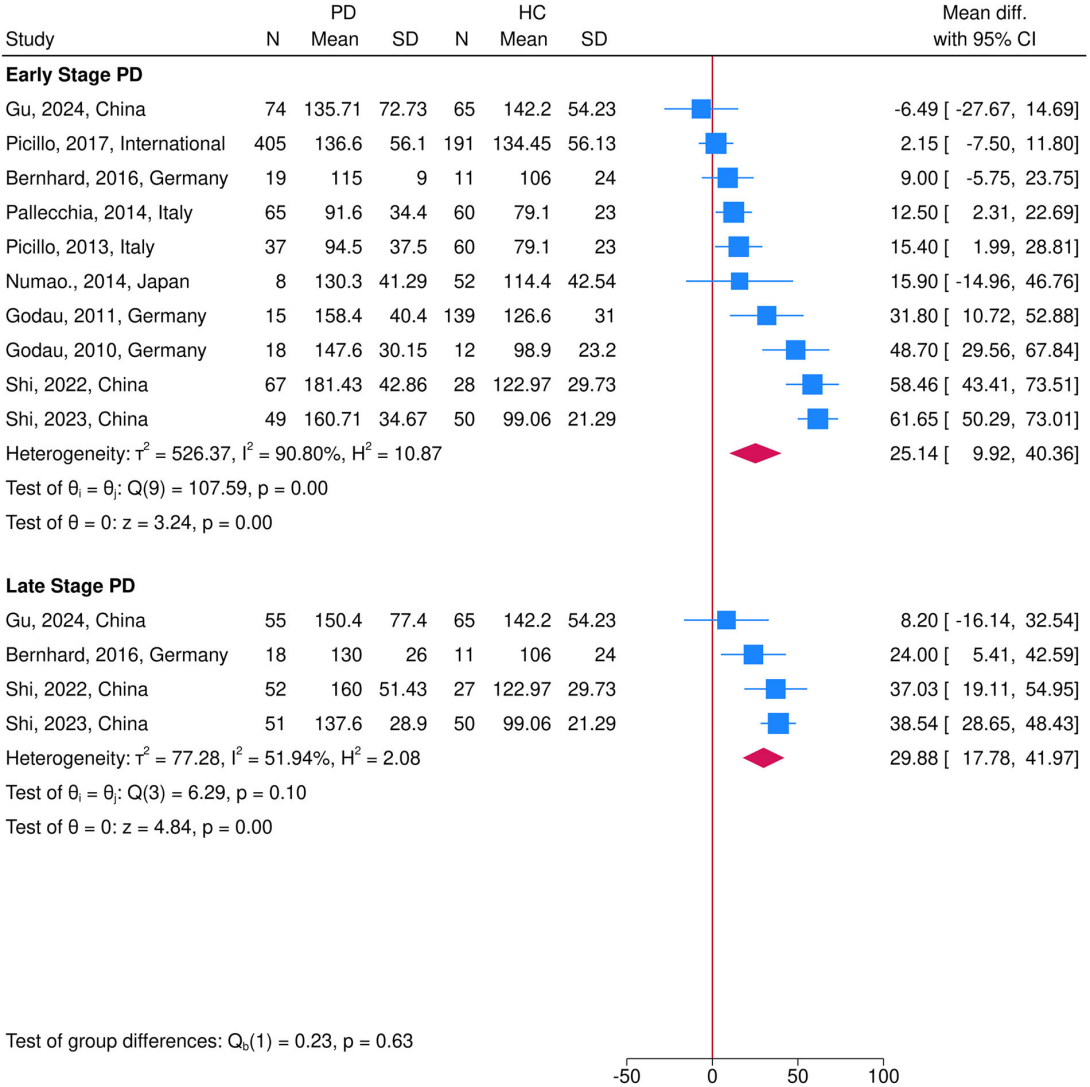
Subgroup analysis of serum IGF‐1 levels comparing early‐stage and late‐stage PD participants versus healthy controls. Effect sizes are presented as mean differences (in ng/mL) calculated using a random‐effects model (early‐stage *I^2^* = 90.80%; late‐stage *I^2^* = 51.94%).

**FIGURE 4:**
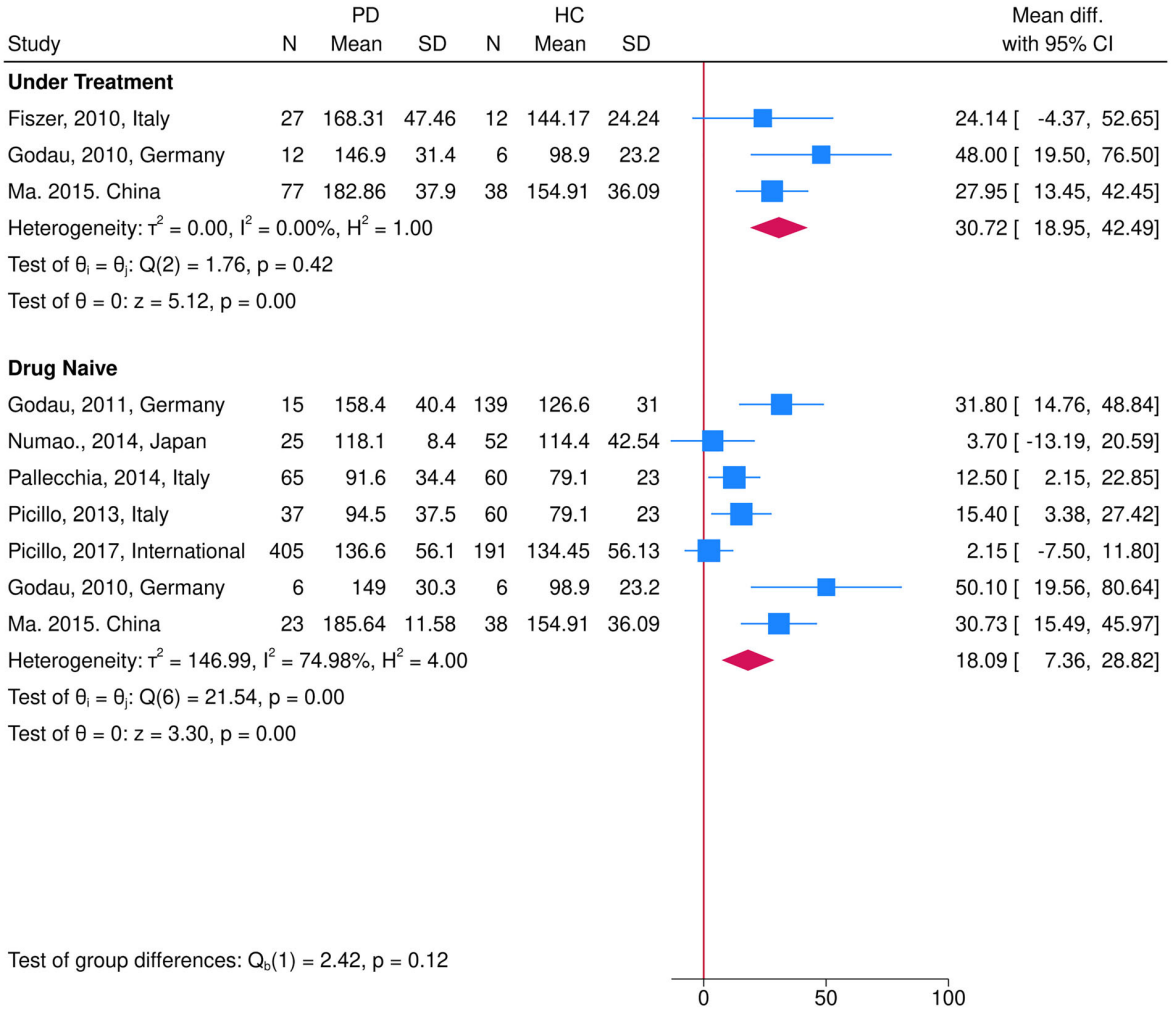
Subgroup analysis comparing drug‐naïve PwPD and those under treatment against healthy controls. Effect sizes are expressed as mean differences (in ng/mL) using a random‐effects model (under treatment *I^2^* = 0.00%; drug‐naïve *I^2^* = 74.98%).

### 3.3 Association Between IGF‐1 and Motor Symptoms

Eight studies (Picillo et al. 2013; Godau et al. 2011; Bernhard et al. 2016; Ma et al. 2015; Numao et al. 2014; Picillo et al. 2017; Shi et al. 2023; Shi et al. 2022) comprising 892 PwPD assessed the association between IGF‐1 and motor symptom severity via UPDRS‐III scores. The studies assessed motor symptoms in the OFF stage with the exception of Ma et al. (2015), who assessed patients in the ON stage and Numao et al. (2014) which lacked information. Whether the original UPDRS questionnaires were used or the MDS revised scales could not be ascertained for a majority of the included studies.

Five of the studies consisting of 413 PwPD were included in the meta‐analysis, which showed that IGF‐1 and UPDRS‐III were inversely correlated (correlation: −0.35; 95% CI: −0.08, −0.53, prediction interval: −0.86, 0.50, *p <* 0.01) with high heterogeneity (*I^2^* = 84%; Figure 5) with very low certainty (Table S4). The main cause of heterogeneity was the study by Ma et al. (2015), which assessed motor symptoms in the ON stage, and whose exclusion reduced the *I^2^* to 34%. Leave‐one‐out analysis also showed the finding to lack robustness (Figure S3). The 3 studies not included in the meta‐analysis were the following:

**FIGURE 5:**
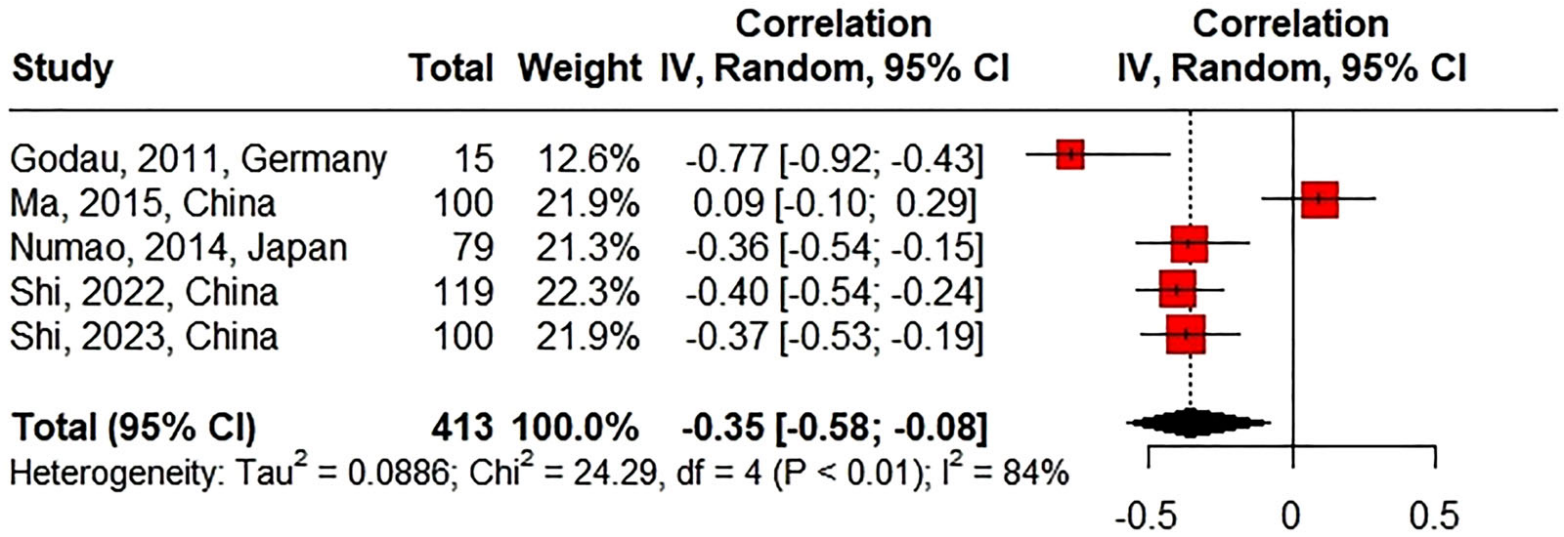
Meta‐analysis of correlation coefficients for the association between serum IGF‐1 and motor symptoms assessed via the Unified Parkinson's Disease Rating Scale (UPDRS‐III) using a random‐effects model (*I^2^* = 84.00).

Bernhard et al. (2016) found that PwPD with high baseline IGF‐1 (> 122 ng/mL) had similar annual UPDRS‐III changes to those with low baseline IGF‐1 after 3.5 years. The two other studies by Picillo et al. (2013) compared UPDRS‐III between quartiles of IGF‐1, with one study finding higher IGF‐1 quartiles had higher UPDRS‐III scores while the other study found no association between the two (Picillo et al. 2017). Overall, the findings were inconsistent.

### 3.4 Association Between IGF‐1 and Non‐Motor Symptoms

Nine studies investigated the association between IGF‐1 levels and non‐motor symptoms in PwPD (Bernhard et al. 2016; Fan et al. 2020; Godau et al. 2010; Gu et al. 2024; Ma et al. 2015; Picillo et al. 2017; Shi et al. 2023; Shi et al. 2022; Suzuki et al. 2019). All nine investigated neuropsychiatric symptoms (Bernhard et al. 2016; Fan et al. 2020; Godau et al. 2010; Gu et al. 2024; Ma et al. 2015; Picillo et al. 2017; Shi et al. 2023; Shi et al. 2022; Suzuki et al. 2019), including cognitive dysfunction assessed in nine studies (Bernhard et al. 2016; Fan et al. 2020; Godau et al. 2010; Gu et al. 2024; Ma et al. 2015; Picillo et al. 2017; Shi et al. 2023; Shi et al. 2022; Suzuki et al. 2019), depression assessed in five studies (Bernhard et al. 2016; Gu et al. 2024; Ma et al. 2015; Shi et al. 2023; Shi et al. 2022), and anxiety assessed in three studies (Gu et al. 2024; Shi et al. 2023; Shi et al. 2022). Four studies assessed sleep disorders (Gu et al. 2024; Ma et al. 2015; Shi et al. 2023; Shi et al. 2022), and only 1 study investigated autonomic symptoms (Ma et al. 2015). Furthermore, three studies assessed all non‐motor symptoms using NMSS (Gu et al. 2024; Ma et al. 2015; Shi et al. 2022). Other symptoms were not investigated. The scales used to evaluate each symptom are presented in Table S6.

For neuropsychiatric symptoms, the pre‐specified 70% concordance criterion was not met for depression, cognitive dysfunction, or anxiety. For depression, two of five studies (40%) reported a positive association between IGF‐1 and symptom severity, whereas three of five (60%) reported no association. For cognitive dysfunction, four of nine studies (44%) reported a positive association and five of nine (56%) reported no association; importantly, none reported an association in the opposite direction, suggesting a consistent but insufficiently supported signal that did not meet the 70% threshold. Similarly, two of three studies (67%) assessing anxiety reported a positive association, while one reported no association. All four studies assessing sleep disorders (100%) reported no association with IGF‐1, meeting the 70% concordance criterion. The single study assessing autonomic dysfunction also reported no association. For overall non‐motor symptom burden, two of three studies (67%) found that higher IGF‐1 levels were associated with lower NMSS scores, whereas Ma et al. (2015) reported no association; therefore, this outcome also did not meet the 70% threshold (Table S6).

### 3.5 Quality Assessment

The majority of our studies were rated as high‐quality based on our assessment, as they demonstrated strong methodology, careful participant selection, and well‐defined outcomes. These studies scored highly on the Newcastle‐Ottawa scale, particularly in the selection and comparability domains, demonstrating minimal risk of bias and reliable findings. However, one study was an exception. The study conducted by Mashayekhi et al. was classified as low quality due to concerns related to the selection domains in the NOS. These concerns indicate potential limitations in the study's design, which may affect the reliability of its findings (Figure S4).

## 4 Discussion

This systematic review and meta‐analysis aimed to assess the potential of IGF‐1 levels as a diagnostic and monitoring biomarker. By pooling data from 16 studies, we demonstrated that PwPD had higher IGF‐1 levels compared with healthy controls. Additionally, we found that factors such as age difference, relative sex ratio, disease duration, and drug‐naïve status did not influence this difference in IGF‐1 levels. We also showed that IGF‐1 levels may be inversely associated with motor symptoms as assessed by UPDRS‐III among PwPD, but more research is required to validate this finding. With regard to non‐motor symptoms, there were insufficient data to perform a meta‐analysis. While two out of three studies assessing non‐motor symptoms using NMSS found an inverse correlation with IGF‐1 levels, we observed no clear direction of effect when examining non‐motor symptoms separately.

Our results are consistent with previous studies on the subject. A 2022 abstract had previously meta‐analyzed the evidence regarding serum IGF‐1 levels as a potential diagnostic biomarker for PD. Similarly to our findings, the report noted that IGF‐1 levels were higher among PwPD compared to healthy controls. However, associations with PD symptoms were not examined in the report (Alwerdani et al. 2022). In addition to its relevance as a biomarker, studies have investigated whether IGF‐1 is causally associated with an increased risk of PD. A longitudinal analysis of 2,116 participants who developed PD during a follow‐up period of 12.6 years revealed a positive linear relationship between IGF‐1 concentrations and risk of PD (Cao et al. 2023). To further investigate the causal nature of this correlation, a two‐sample Mendelian randomization was subsequently conducted. In a primary causal effects model using the inverse‐variance weighted method, IGF‐1 levels were significantly associated with PD risk, with minimal risk of bias from horizontal pleiotropy. This suggests that IGF‐1 levels may increase the susceptibility to the development of PD (Xu et al. 2024).

Beyond PD, IGF‐1 levels have also been investigated in other neurological conditions and psychiatric disorders. In Alzheimer's disease (AD), a meta‐analysis of 17 studies involving more than 2,200 individuals found lower IGF‐1 levels in patients, although the difference from healthy controls was not statistically significant, but significantly lower levels were observed in larger studies and in cerebrospinal fluid (Xu et al. 2021). However, prospective cohort studies have associated lower serum IGF‐1 levels with an increased risk of developing AD (Westwood et al. 2014). In ischemic stroke, evidence from 17 studies including 2,277 patients showed that higher serum IGF‐1 levels were significantly associated with a lower risk of stroke and greater functional improvement after stroke (Li et al. 2022). In contrast, a review of 14 studies involving 285 patients with bipolar disorder (BD) and 503 with major depressive disorder (MDD) found significantly higher peripheral IGF‐1 levels in both groups than in healthy controls (Chen et al. 2020). Overall, IGF‐1 appears to be altered in stroke, BD, and MDD, and these changes may overlap with those observed in PD. However, the specificity of IGF‐1 as a biomarker for PD is limited.

At the cellular and molecular level, preclinical evidence supports a direct role of IGF signaling in dopaminergic neuron function and survival. IGF‐1 and IGF‐1R are expressed in midbrain dopaminergic neurons, and locally derived IGF‐1 regulates neuronal firing, striatal dopamine levels, locomotion, and motor learning, indicating a functional role distinct from circulating liver‐derived IGF‐1 (Pristerà et al. 2019). Reduced IGF‐1/IGF‐1R signaling increases dopaminergic vulnerability to MPTP and 6‐OHDA, whereas IGF‐1 treatment attenuates nigral neuronal and striatal terminal loss through pathways involving PI3K/Akt, ERK, mTOR, and inhibition of GSK‐3β‐mediated pro‐death signaling (Nadjar et al. 2009; Quesada and Micevych 2004; Ayadi et al. 2016). IGF‐1 signaling also modulates autophagy, α‐synuclein‐related changes, oxidative stress, mitochondrial function, NRF2/HO‐1 activity, and GDNF production in MPTP/MPP+, rotenone, and dopaminergic cellular models (Kim et al. 2014; Wang et al. 2020; Zheng et al. 2023). Aging may further compromise this trophic system, as IGF‐1 levels decline in the substantia nigra and central IGF‐1R expression decreases with age (Ogundele et al. 2018; Muñoz et al. 2018). These findings raise an important mechanistic question in relation to the elevated serum IGF‐1 observed in PwPD: because neuronal and circulating IGF‐1 represent distinct compartments, higher peripheral IGF‐1 does not necessarily imply preserved or enhanced IGF signaling within the nigrostriatal system (Pristerà et al. 2019). However, the available preclinical studies do not directly demonstrate a reduction in endogenous IGF‐1 or IGF‐1R signaling at a defined presymptomatic stage before overt motor impairment; therefore, the temporal relationship between central IGF signaling and dopaminergic degeneration remains unresolved. This central–peripheral dissociation supports the need for longitudinal studies incorporating paired serum and CSF IGF‐1 measurements across disease stages.

The paradox of elevated IGF‐1 increasing PD risk while being inversely linked to PD symptoms has sparked speculation regarding the underlying mechanisms. IGF‐1 is a key mitogenic factor essential for brain growth and development, promoting neuron viability, the extension of neuronal projections, the development of oligodendrocytes, and the formation of myelin (D'Ercole et al. 1996). Studies have found that traumatic brain injuries upregulate the levels of IGF‐1, possibly as an attempt at endogenous neuroprotection or repair (Kizhakke Madathil et al. 2010). Furthermore, data suggests that treatment with IGF‐1 assists in the process of repair after nerve injuries (Apel et al. 2010). Based on these findings, one possible theory is that the elevated IGF‐1 levels observed among PwPD represent a compensatory response to neuronal injury occurring before and during the clinical manifestation of the disease. Under this hypothesis, the inverse association between IGF‐1 levels and motor symptom severity could reflect an insufficient or impaired compensatory response in individuals with greater disease burden, potentially contributing to greater neuronal injury and symptom severity. A similar pattern can be observed regarding ischemic stroke, as serum IGF‐1 levels are inversely correlated with neurological deficits and disability following a stroke (Hayes et al. 2021). However, this compensatory mechanism remains hypothetical, as the predominantly cross‐sectional evidence included in our review cannot establish the temporal direction or causality of these associations. Reverse causation represents an important alternative explanation, whereby greater PD‐related disease burden or systemic changes accompanying disease severity may influence circulating IGF‐1 levels rather than reduced IGF‐1 contributing to disease progression. Moreover, neither disease stage nor disease duration significantly moderated the difference in IGF‐1 levels between PwPD and healthy controls in our analyses, providing no clear evidence for a progressive failure of compensation. Therefore, although a compensatory role for IGF‐1 is biologically plausible, our findings should be interpreted as hypothesis‐generating rather than evidence of a causal mechanism. Longitudinal studies assessing changes in IGF‐1 alongside disease progression are needed to distinguish between these competing explanations. Furthermore, IGF‐2 may represent an additional component of the neuroprotective response to neuronal injury. Experimental evidence indicates that IGF‐2 can reduce α‐synuclein accumulation and attenuate dopaminergic neuronal loss in preclinical models of PD (Arcos et al. 2023). Given that IGF‐2 can activate signaling pathways that overlap with those activated by IGF‐1, changes in IGF‐2 activity could theoretically modify the relationship between circulating IGF‐1 levels and clinical manifestations (Beletskiy et al. 2021). Consistent with a potential role for IGF‐2 in human PD, a recent case‐control study involving 43 patients with idiopathic PD and 41 controls found significantly higher serum IGF‐2 levels in patients, whereas IGF‐1 levels did not differ significantly between groups (Cakir et al. 2025). However, whether IGF‐2 compensates for altered IGF‐1 signaling in human PD and whether this mechanism contributes to the heterogeneous associations observed in this review remains unknown and merits further investigation.

Given the role of IGF‐1 in dopaminergic neuronal survival, its therapeutic potential in PD warrants consideration. Despite promising preclinical findings described (Nadjar et al. 2009; Quesada and Micevych 2004; Ayadi et al. 2016), translating IGF‐1 neuroprotection into a treatment for PD remains challenging. Systemic administration may not ensure adequate central target engagement and carries risks such as hypoglycemia (Bang et al. 2024). Clinical trials in other neurodegenerative disorders illustrate these limitations: in ALS, randomized trials of recombinant IGF‐1 yielded inconsistent results, with subsequent studies failing to confirm the benefits reported initially (Beauverd et al. 2012). Although ALS differs pathophysiologically from PD, these findings underscore the need to establish the efficacy and safety of IGF‐1‐based interventions specifically in PD. Future studies should determine whether targeted delivery or modulation of IGF‐1 signaling can preserve nigrostriatal neurons and improve functional outcomes, while clarifying the optimal treatment window and potential adverse effects.

Our study has several limitations. First, our analysis comparing IGF‐1 levels between PwPD and healthy controls was marked by a significant level of heterogeneity. Although we strived to address this issue by conducting multiple subgroup analyses and meta‐regressions, none of the investigated confounders were found to exert a significant influence on our results, including age difference, relative sex ratio, drug‐naïve status, disease duration, and disease stage. Further studies with larger sample sizes are needed to better investigate the sources of heterogeneity observed in our results. Second, our analysis of motor symptoms was also limited by a high degree of heterogeneity. However, we were not able to fully examine this heterogeneity due to the small number of studies, although excluding the study that assessed motor symptoms in the ON stage significantly reduced heterogeneity. Other factors such as the type of UPDRS questionnaire may have also played a role. In addition, the small number of studies examining non‐motor symptoms did not allow us to draw quantitative conclusions. We suggest that more in‐depth research be done with regard to the symptoms of PD and IGF‐1 to remedy this limitation. Third, we only examined the peripheral IGF‐1 levels in relation to PD, while the CSF levels have not been examined and merit further research. None of the included studies assessed IGF‐1 levels in individuals with prodromal or at‐risk PD phenotypes. Consequently, it remains unknown whether alterations in IGF‐1 occur before the onset of clinically manifest PD or emerge only after diagnosis. This limits conclusions regarding the potential value of IGF‐1 as an early‐detection or disease‐monitoring biomarker and highlights the need for longitudinal studies in well‐characterized prodromal and genetically at‐risk populations. Fourth, estrogen levels and estrogen replacement therapy can affect IGF‐1 levels (Sonnet et al. 2007). While most of the included studies had matched for sex and meta‐regressions were non‐significant for sex, hormonal therapy among female participants may have confounded some of the reported findings. Lastly, dopaminergic therapy and nutritional status may have confounding effects on IGF‐1 levels (Fouad et al. 2024; Cylke‐Falkowska et al. 2026), yet this potential source of heterogeneity has been sparsely reported in the literature but may explain some of the observed between study heterogeneity.

Peripheral IGF‐1 is generally higher among PwPD compared with HC, while possibly correlating inversely with PD motor symptoms. While this demonstrates its potential as a monitoring biomarker for PD, the small difference between PwPD and healthy controls may not allow for its adoption as a diagnostic biomarker. Further studies are needed to examine the heterogeneity of these correlations and to explore their underlying mechanisms. Clarifying these mechanisms could also support the development of IGF‐1–based therapeutic strategies. In addition, the association between IGF‐1 and non‐motor symptoms of PD is under‐investigated by the current literature and merits further research.

## Author Contributions

**Sajjad Mahdavi**: conceptualization, project administration, investigation. **Shahryar Rajai Firouzabadi**: methodology, formal analysis, validation**. Ida Mohammadi**: methodology, writing – original draft**. Soroush Oraee**: writing – original draft, writing – review and editing. **Mohammadreza Alinejadfard**: writing – original draft, writing – review and editing. **Seyed Ali Mofidi**: writing – original draft, writing – review and editing. **Mehri Salari**: conceptualization, project administration. **Ehsan Sharifipour**: conceptualization, project administration, investigation. all authors have read and agreed to the published version of the manuscript.

## Funding

The authors have nothing to report.

## Ethics Statement

This study follows the ethical guidelines of the Shahid Beheshti University of Medical Sciences Ethical Committee (IR.SBMU.MSP.REC.1404.057). Informed patient consent was not necessary for this work. We confirm that we have read the Journal's position on issues involved in ethical publication and affirm that this work is consistent with those guidelines.

## Conflicts of Interest

The authors declare no competing interests.

## Supporting information




**Supplemental Table 1**: Search strategies for different databases
**Supplemental Table 2**: Characteristics of the included studies.
**Supplemental Table 3**: Outcomes assessed in each study.
**Supplemental Table 4**: Certainty of evidence assessment
**Supplemental Table 5**: Meta‐regressions of age difference, relative sex ratio, and disease duration for the mean difference of IGF‐1 meta‐analysis.
**Supplemental Table 6**: Effect direction plot for non‐motor symptoms.
**Supplemental Figure 1**: Funnel plot of standard error by the difference in means of studies that compared serum IGF‐1 levels between PwPD and healthy controls.
**Supplemental Figure 2**: Sensitivity analysis of studies that compared serum IGF‐1 levels between PwPD and healthy controls
**Supplemental Figure 3**: Quality assessment of included studies. A) Cohort studies B) Cross‐sectional studies C) Case‐control studies

## Data Availability

Data sharing is not applicable, as no datasets were generated or analyzed.
