# Supplementary material for: Serum Insulin‐Like Growth Factor‐1 (IGF‐1) in Parkinson's Disease: A Systematic Review and Meta‐Analysis
Source: Brain Behav. 2026 Nov 5;16(11):e71872. doi: 10.1002/brb3.71872 (PMC13740353; doi:10.1002/brb3.71872)
Supplement: Supplementary file 1 [file BRB3-16-e71872-s001.docx]

**Supplementary Material 1**

Serum Insulin-Like Growth Factor-1 (IGF-1) In Parkinson’s Disease: A Systematic Review and Meta-Analysis

**Supplementary Materials – Index:**

| **Supplementary Figures and Tables** |  |
| --- | --- |
| Table S1 | *page.2* |
| Table S2 | *page.3* |
| Table S3  Table S4  Table S5  Table S6 | *page.6*  *page.8*  *page.9*  *page.10* |
| Figure S1 | *page.11* |
| Figure S2  Figure S3 | *page.12*  *page.13* |
| Figure S4 | *page.14* |

| Table S1: Search strategies fot different databases |  |
| --- | --- |
| Syntax | N |
| Pubmed/MEDLINE | |
| ((((((((Insulin-Like Growth Factor I[Title/Abstract]) OR (IGF-1[Title/Abstract])) OR (IGF-I[Title/Abstract])) OR (Insulin-Like Somatomedin Peptide I[Title/Abstract])) OR (Insulin Like Growth Factor I[Title/Abstract])) OR (Somatomedin C[Title/Abstract])) OR (Insulin Like Somatomedin Peptide I[Title/Abstract])) OR (IGF-I-SmC[Title/Abstract])) AND ((((((((((((Parkinson Disease[Title/Abstract]) OR (Idiopathic Parkinson's Disease[Title/Abstract])) OR (Lewy Body Parkinson's Disease[Title/Abstract])) OR (Parkinson's Disease, Idiopathic[Title/Abstract])) OR (Parkinson's Disease, Lewy Body[Title/Abstract])) OR (Paralysis Agitans[Title/Abstract])) OR (Parkinson's Disease[Title/Abstract])) OR (Idiopathic Parkinson Disease[Title/Abstract])) OR (Lewy Body Parkinson Disease[Title/Abstract])) OR (Primary Parkinsonism[Title/Abstract])) OR (Parkinsonism, Primary[Title/Abstract])) OR (Parkinson Disease, Idiopathic[Title/Abstract])) | 139 |
| Embase | |
| (((((((('insulin-like growth factor i':ti,ab,kw) OR ('igf-1':ti,ab,kw)) OR ('igf-i':ti,ab,kw)) OR ('insulin-like somatomedin peptide i':ti,ab,kw)) OR ('insulin like growth factor i':ti,ab,kw)) OR ('somatomedin c':ti,ab,kw)) OR ('insulin like somatomedin peptide i':ti,ab,kw)) OR ('igf-i-smc':ti,ab,kw)) AND (((((((((((('parkinson disease':ti,ab,kw) OR ('idiopathic parkinson`s disease':ti,ab,kw)) OR ('lewy body parkinson`s disease':ti,ab,kw)) OR ('parkinson`s disease, idiopathic':ti,ab,kw)) OR ('parkinson`s disease, lewy body':ti,ab,kw)) OR ('paralysis agitans':ti,ab,kw)) OR ('parkinson`s disease':ti,ab,kw)) OR ('idiopathic parkinson disease':ti,ab,kw)) OR ('lewy body parkinson disease':ti,ab,kw)) OR ('primary parkinsonism':ti,ab,kw)) OR ('parkinsonism, primary':ti,ab,kw)) OR ('parkinson disease, idiopathic':ti,ab,kw)) | 186 |
| Web of Science | |
| TS=("Insulin-Like Growth Factor I" OR "IGF-1" OR "IGF-I" OR "Insulin-Like Somatomedin Peptide I" OR "Insulin Like Growth Factor I" OR "Somatomedin C" OR "Insulin Like Somatomedin Peptide I" OR "IGF-I-SmC")  AND  TS=("Parkinson Disease" OR "Idiopathic Parkinson's Disease" OR "Lewy Body Parkinson's Disease" OR "Parkinson's Disease, Idiopathic" OR "Parkinson's Disease, Lewy Body" OR "Paralysis Agitans" OR "Parkinson's Disease" OR "Idiopathic Parkinson Disease" OR "Lewy Body Parkinson Disease" OR "Primary Parkinsonism" OR "Parkinsonism, Primary" OR "Parkinson Disease, Idiopathic") | 159 |
|  |  |

Table S2 Characteristics of the included studies.

| Author, Year, Country | Study design, Follow-up | Source of data | Fasting or non-fasting sample, Method of assessment | Number of participants | | | PD characteristics | | | | Age*,  Gender** | | Adjustment for confounding factors | |
| --- | --- | --- | --- | --- | --- | --- | --- | --- | --- | --- | --- | --- | --- | --- |
|  |  |  |  | T | PD | HC | Drug Naïve | Early or Late stage | | Disease duration* | PD | HC | |  |
| Bernhard, 2016, Germany (30) | Cohort, 3.5 years | MODEP study | Fasting, IRMA | 59 | 37 | 22 | No | Mixed | maximum 8 years | | 64 (7), 59% | 64 (7), 59% | | Matched for DM, TD, cancer and beta blocker medication |
| Fan, 2020, New Zealand (31) | Cross sectional | Local EMR | Non-fasting, ELISA | 201 | 178 | 23 | No | Mixed | 11.54 (0.7) | | 72.6 (6.7), 70% | 74.5 (1.4), 66% | | Excluded major  medical illness  , Depression, major  psychiatric disorders, dementia |
| Federico, 1:999, Italy (32) | Cross sectional | Local EMR | Fasting, IRMA | 20 | 10 | 10 | No | Mixed | 4.7 (3.2) | | 60.5 (5.3). 50.0% | 60.3 (5.3), 60.0% | | NP |
| Fiszer, 2010, Poland (33) | Cross sectional | Local EMR | NP, IRMA | 39 | 27 | 12 | No | Mixed | 7.3 (4.1) | | 61.7 (8.7), 48% | 58.6 (8.6), 25% | | Excluded DM, TD, cancer and infectious  diseases |
| Godau, 2010, Germany (34) | Cohort, 6 months | Local EMR | Fasting, CLIA | 30 | 18 | 12 | Mixed | Early | 2.7 (2.4) | | 66.3 (6.33), 77% | 66 (6.0), 75% | | Excluded DM, TD, obesity, cancer, corticosteroid, beta blocker |
| Godau, 2011, Germany (17) | Cross sectional | PRIPS study | Fasting, IRMA | 154 | 15 | 139 | Yes | Early | NP | | 69.0 (8.3), 60% | 60.6 (6.6), 71% | | Excluded DM, TD, obesity, cancer, corticosteroid, beta blocker |
| Gu, 2024, China (35) | Cohort, NP | Local EMR | Fasting, CLIA | 259 | 129 | 130 | No | Mixed | 5 (3) | | 62.4 (9.2) | 62.2 (8.18) | | Excluded TD and cancer  Matched for DM |
| Ma, 2015, China (36) | Cross sectional | Local EMR | Fasting, ELISA | 176 | 100 | 76 | Mixed | Mixed | 4.4 (3.9) | | 70.1 (11.0), 67% | 67.8 (8.6), 59.2 | | Excluded DM, TD, obesity, cancer, corticosteroid, beta blocker |
| Mashayekhi, 2010, Iran (37) | Cross sectional | Local EMR | NP, ELISA | 76 | 38 | 38 | No | NP | NP | | NP | NP | | Excluded DM, cancer, infectious disease |
| Numao, 2014, Japan (38) | Case Control | Local EMR | Fasting, IRMA | 131 | 79 | 52 | Mixed | Mixed | 5.4 (5.3) | | 68.1 (9.7), 44% | 65.6 (8.6), 55% | | Excluded DM, TD, obesity, cancer, corticosteroid, dementia, psychiatric disorders |
| Pellecchia, 2014, Italy (39) | Cohort, 2 years | Local EMR | Fasting, ELISA | 125 | 65 | 50 | Yes | Early | 1.2 (0.69) | | 59.7 (8.3) , 60% | 58.8 (7.2) 60% | | Excluded disorders interfering with serum IGF-1 levels. |
| Picillo, 2013, Italy (16) | cohort, 2 years | Local EMR | Fasting, ELISA | 97 | 37 | 60 | Yes | Early | 1.2 (0.5) | | 58.2 (9), 60% | 58.8 (7.2), 60% | | Excluded DM, TD, obesity, cancer, corticosteroid, beta blocker |
| Picillo, 2017, International (40) | Cohort, 5 years | PPMI | Fasting, ELISA | 596 | 405 | 191 | Yes | Early | 0.5 (0.5) | | 61.2 (9.8), 65.2% | NP | | Excluded DM, TD, obesity, cancer, corticosteroid |
| Schaefer, 2018, Germany (41) | Case Control | Local EMR | Fasting, CLIA | 20 | 10 | 10 | No | Mixed | 8.1 (5.7) | | 55.2 (8.0) | NP | | Excluded DM, TD, obesity, cancer, beta blocker |
| Shi, 2022, China (43) | Cross sectional | Local EMR | Fasting, CLIA | 174 | 119 | 55 | No | Mixed | 5 (4.8) | | 62.7 (7.5), 45.% | 62.6 (6.7), 57% | | Excluded CVD, infectious disease |
| Shi, 2023, China (42) | Cross sectional | Local EMR | Fasting, CLIA | 200 | 100 | 100 | No | Mixed | NP | | 63.4 (9.0), 56% | 63.0 (6.4), 52% | | Excluded CVD, infectious disease |
| Suzuki, 2019, Japan (44) | Cross sectional | Local EMR | Non-fasting, IRMA | 73 | 73 | NA | No | Mixed | 4.1 (4.3) | | 69.4 (9.5), 73% | NP | | Excluded DM, TD, obesity and cancer |
| Tuncel. 2009, Turkey (45) | Cross sectional | Local EMR | Fasting, CLIA | 50 | 25 | 25 | No | Mixed | 4*.*9 (3*.*1) | | 67*.*9 (9*.*4), 80% | 64*.*3 (80), 80% | | Excluded stroke and dementia, |

^*: reported as mean (standard deviation); **: reported as male %
Abbreviations: MODEP: modeling epidemiological data to study parkinson disease progression / EMR: electronic medical records / PRIPS: prospective validation of risk Factors for parkinsonian syndromes / PPMI: parkinson's progression markers initiative / SD: standard deviation / CLIA: chemiluminescence immunoassay / ELISA: enzyme-linked immunosorbent assay / IRMA: immunoradiometric assay / NP: not provided / T: total / PD: parkinson’s disease / HC: healthy control / NA: not applicable/ DM: diabetes mellitus/ TD: Thyroid dysfunction^

Table S3 Outcomes assessed in each study.

| Study | Comparison of IGF-1 in PwPD vs HC | Association with motor symptoms | Association with non-motor symptoms |
| --- | --- | --- | --- |
| Bernhard, 2016, Germany (30) | ✓ | ✓ | ✓ |
| Fan, 2020, New Zealand (31) | ✓ | - | ✓ |
| Federico, 1999, Italy (32) | ✓ | - | - |
| Fiszer, 2010, Poland (33) | ✓ | - | - |
| Godau, 2010, Germany (34) | ✓ | - | ✓ |
| Godau, 2011, Germany (17) | ✓ | ✓ | - |
| Gu, 2024, China (35) | ✓ | - | ✓ |
| Ma, 2015, China (36) | ✓ | ✓ | ✓ |
| Mashayekhi, 2010, Iran (37) | ✓ | - | - |
| Numao, 2014, Japan (38) | ✓ | ✓ | - |
| Pellecchia, 2014, Italy (39) | ✓ | - | - |
| Picillo, 2013, Italy (16) | ✓ | ✓ | - |
| Picillo, 2017, International (40) | ✓ | ✓ | ✓ |
| Schaefer, 2018, Germany (41) | ✓ | - | - |
| Shi, 2022, China (43) | ✓ | ✓ | ✓ |
| Shi, 2023, China (42) | ✓ | ✓ | ✓ |
| Suzuki, 2019, Japan (44) | - | - | ✓ |
| Tuncel. 2009, Turkey (45) | ✓ | - | - |

| Table S4: Certainty of evidence assessment | | | | | | | | | | | |
| --- | --- | --- | --- | --- | --- | --- | --- | --- | --- | --- | --- |
| **Certainty assessment** | | | | | | | **№ of patients** | | **Effect (CI 95%)** | **Certainty** | **Importance** |
| **№ of studies** | **Study design** | **Risk of bias** | **Inconsistency** | **Indirectness** | **Imprecision** | **Other considerations** | **Parkinson's Disease** | **Healthy Controls** |  |  |  |
| **Serum IGF-1 in PD vs HC Patients** | | | | | | | | | | | |
| 17 | non-randomised studies | not serious | serious^a^ | not serious | not serious | strong association | 1392 | 1015 | MD: **21.3 ng/ml**  (11.49 to 31.12) | ⨁⨁◯◯ Low^a^ |  |
| **Association between IGF-1 and Motor Symptoms** | | | | | | | | | | | |
| 8 | non-randomised studies | not serious | serious^a^ | not serious | not serious | publication bias strongly suspected^b^ | 892 | 0 | Correlation: **-0.35**  (-0.58 to -0.08) | ⨁◯◯◯ Very low^a,b^ |  |

**CI:** confidence interval; **MD:** mean difference

#### Explanations

a. I^2^> 50% was considered as a serious inconsistency.

b. The publication bias analysis was not conducted due to limitations in the number of included studies.

Table S5 : Meta-regressions of age difference, relative gender ratio, and disease duration for the mean difference of IGF-1 meta-analysis.

| Confounder | Number of studies | Number of participants (PD vs HC) | Coefficient (p-value) | R^2^ |
| --- | --- | --- | --- | --- |
| Age difference | 14 | 1,715 (939 vs 776) | 0.871 (0.709) | 0% |
| Relative gender ratio | 14 | 1,715 (939 vs 776) | 1.157 (0.956) | 0% |
| Disease duration | 13 | 2,111 (1,244 vs 867) | -0.512 (0.776) | 0% |

Table S6 Effect direction plot for non-motor symptoms.

| **Study** | **Neuropsychiatric symptoms** | | | **Sleep  disorders** | **Autonomic  symptoms** | **Non-motor symptoms scale** |
| --- | --- | --- | --- | --- | --- | --- |
|  | Depression^a^ | Cognitive  dysfunction | Anxiety |  |  |  |
| Bernhard, 2016, Germany (30) | ◄►^b^ | ◄►^a^ |  |  |  |  |
| Fan, 2020, New Zealand (31) |  | ◄►^c, d^ |  |  |  |  |
| Godau, 2010, Germany (34) |  | ◄►^a^ |  |  |  |  |
| Gu, 2024, China (35) | ◄►^e^ | ▲^c^ | ◄►^f^ | ◄►^g, h^ |  | ▲^i^ |
| Ma, 2015, China (36) | ◄►^e^ | ▲^a^ |  | ◄►^j^ | ◄►^k^ | ◄►^i^ |
| Picillo, 2017, International (40) |  | ◄►^c^ |  |  |  |  |
| Shi, 2022, China (43) | ▲^e^ | ▲^a^ | ▲^f^ | ◄►^h^ |  | ▲^i^ |
| Shi, 2023, China (42) | ▲^e^ | ▲^a^ | ▲^f^ | ◄►^h^ |  |  |
| Suzuki, 2019, Japan (44) |  | ◄►^a^ |  |  |  |  |

^Legend:^

^Effect direction: upward arrow ▲= positive health impact, downward arrow ▼= negative health impact, sideways arrow ◄►= no change/mixed effects/conflicting findings
Sample size: Large arrow ▲ >100; medium arrow ▲ 50-100; small arrow ▲ <50^

^a: MMSE, b: BDI-II, c: MoCA, d: Global Z score, e: HAMD-17, f: HAMA-14, g: ESS, h: PSQI, i: NMSS, j: RBDSQ, k: SCOPA-AUT^

**Figure S1.** Funnel plot of standard error by the difference in means of studies that compared serum IGF-1 levels between PD patients and healthy controls.

**Figure S2.** Sensitivity analysis of studies that compared serum IGF-1 levels between PD patients and healthy controls.

**Figure S3.** Sensitivity analysis of studies that examined the association between serum IGF-1 levels and motor symptoms.

**Figure S4.** Quality assessment of included studies. A) Cohort studies B) Cross-sectional studies C) Case-control studies
